# Enhancing french basil growth through synergistic Foliar treatment with copper nanoparticles and *Spirulina* sp.

**DOI:** 10.1186/s12870-024-05153-x

**Published:** 2024-06-07

**Authors:** Heba Mahmoud Elbanna, Osama Konsowa Ahmed, Sayed Abdel-Kader Fayed, Khaled Abdel-Moneim Hammam, Rania Saber Yousef

**Affiliations:** 1 Medicinal and Aromatic Plants Research Department, Horticulture Research Institute, Agriculture Research Center, Giza, Egypt; 2 Biochemistry Department, Faculty of Agriculture, Cairo University, Giza, Egypt

**Keywords:** Green synthesizer, Essential oils, Antioxidant activity, Spirulina extract

## Abstract

**Background:**

This study investigates a novel idea about the foliar application of nanoparticles as nanofertilizer combined with a natural stimulant, blue-green algae *Spirulina platensis* L. extract, as a bio-fertilizer to achieve safety from using nanoparticles for enhancement of the growth and production of the plant. Thus, this experiment aimed to chemically synthesize copper nanoparticles via copper sulfate in addition to evaluate the impact of CuNPs at 500, 1000, and 1500 mg/L and the combination of CuNPs with or without microalgae extract at 0.5, 1, and 1.5 g/L on the morphological parameters, photosynthetic pigments accumulation, essential oil production, and antioxidant activity of French basil.

**Results:**

The results revealed that foliar application of CuNPs and its interaction with spirulina extract significantly increased growth and yield compared with control, the treatments of 1000 and 1500 mg/L had less impact than 500 mg/L CuNPs. Plants treated with 500 mg/L CuNPs and 1.5 g/L spirulina extract showed the best growth and oil production, as well as the highest accumulation of chlorophylls and carotenoids. The application of CuNPs nanofertilizer caused a significant increase in the antioxidant activity of the French basil plant, but the combination of CuNPs with spirulina extract caused a decrease in antioxidant activity.

**Conculosion:**

Therefore, foliar application of natural bio-fertilizer with CuNPsis necessary for obtaining the best growth and highest oil production from the French basil plant with the least damage to the plant and the environment.

## Background

The basil plant is one of the most important and economical annual herbaceous plants in the *Lamiaceae* family [1, 2]. Basil (*Ocimum basilicum* L. var. Grand Vert) is a variety that is well adapted to various growing conditions; furthermore, it has been cultivated for the highly important value of its volatile oil [3], which has antimicrobial, diuretic, analgesic, anti-inflammatory, antiviral, and antioxidant properties [4]. Egypt, Spain, Hungary, and France are the world’s main producers of basil [5]. Not only does basil have traditional uses, but it also has super-effective anti-kidney disorders, colds, and diarrhea due to its richness in bioactive compounds, in addition to protecting against cardiovascular diseases and nerve pains [6]. As well as, researchersproved the biological activities of basil essential oil and aqueous extracts as antidiabetic and dermatoprotective effects [7].

The main compounds of basil essential oil are phenylpropanoids and terpenes as a complex structure; therefore, basil is unique among the best aromatic plants in the world in folk medicine and the food, perfume, and cosmetics industries [8]. According to [9], the essential oils extracted from basil parts are abundant with volatile organic compounds thus, basil essential oil is valuable for food and pharmaceutical industries with strong market demand. Furthermore, another research [10] discussed that *Ocimum basilicum* L. is thought to have immunomodulatory and antioxidant properties. Linalool, 1,8-cineole, methylchavicol (estragole), and eugenol are the most significant chemical components of basil essential oil [11]. Additionally [12], documented that a basil ethanolic extract containing eugenol exhibited lethal effects on human laryngeal carcinoma cells. Many studies reported that the essential oil content of the herb is about 0.04–0.70% [13]. Also, basil herbs contain flavonoids, including quercetin and kaempferol glycosides, as well as phenolic acids with a majority of caffeic acid [14]. Several species have also been defined as linked with essential oil constituents (chemotypes) [15].

Nanotechnology has received much attention because of its distinctive properties and many applications in various fields furthermore, it is a new approach towardincreasing agricultural production with premium quality, environmental safety, biological support, and financial stability also, eco-friendly technology is becoming increasingly important in modern agricultural applications as an alternative to traditional fertilizers and pesticides so, nanotechnology offers an alternative solution to overcome the disadvantages of conventional agriculture therefore, recent developments in using nanoparticles in agriculture should be studied [16]. Nanoparticles (NPs) are tiny molecules with a small size range of 1–100 nm with different physiochemical properties than bulk materials [17]. Based on the previous study [18], NPs were improved their physical, chemical, and biological properties and functions due to their expanded surface area-to-volume ratio. Nano-fertilizers provide some nutrients in nanoform, enhancing plant growth and production [19].

The advantage of foliar fertilizers is that they avoid the toxicity that happens with soil application of the microelements [20]. Therefore, using foliar sprays with fertilizers containing microelements such as manganese (Mn), iron (Fe), Zn, B, and copper (Cu), which affect plants effectively and rapidly, has a great response [21]. The evaluated studies examined the effects of several metal nanoparticles (NPs) on a variety of crop plants, including copper, silver, zinc, gold, and titanium, and they noted significant morphological changes in the plants [22]. Copper-based nanoparticles are among the most promising nanomaterials to replace conventional agrochemicals hence, most of the studies have been performed with the three Cu-based NPs, CuONPs have been shown to have some advantages compared to their micrometer-sized counterparts but all nanomaterials, the response of plants is linked to varietal differences, applied concentrations, application mode, and the culture medium [23].

Recently, biofertilizer application has led to crop sustainability with eco-friendly microalgae [24]. One of the vital cyanobacteria is the blue-green microalga, *Spirulina platensis* extracts increase plant growth, seed germination, fruit production, and flowering by enhancing mineral nutrient utilization also, spirulina is rich in organic and inorganic nutrients [25, 26]. According to [27], spirulina is applied to numerous crops using a variety of application techniques, either alone or in combination with other organic fertilizers, achieving super-environmental agriculture.Therefore, for cost-effective fennel production, utilizing algal extracts of *Spirulina platensis* and compost tea as biological stimulants along with only 75% of the recommended dose of nitrogen fertilizer [28], previous studies reported that foliar sprays of various biostimulators in nanoform affected the properties and oil yield of marjoram and basil plants [29–31]. Considering novel findings on the positive effects of nanofertilizers by [3, 32, 33] for basil plants [34], chili, and [35] peppermint. Additionally, high efficiency in enhancing photosynthesis [36] and antioxidants [37]; on the contrary, high concentrations of CuNPs in cucumber may also lead to phytotoxicity in lettuce and *Oryzasativa*, according to [38, 39]. At the same time, cyanobacteria can decrease the stress faced by recent agriculture [40].

The purpose of our study was to investigate the response of French basil growth, yield quantity and chemical composition of essential oil, pigment content, quality, and antioxidant enzymeactivity as a result of a modern tactic with foliar application of nano fertilizer (copper nanoparticles) individually and combined with biofertilizer (spirulina extract) at various concentrations.

## Materials and methods

This study was carried out during two successive seasons (2020 and 2021) of pot experiments in natural field conditions on the farm of the Medicinal and Aromatic Plants Department, Horticulture Research Institute, Agriculture Research Center, Giza, Egypt.

### Synthesis of copper nanoparticles

**Fig. 1:**
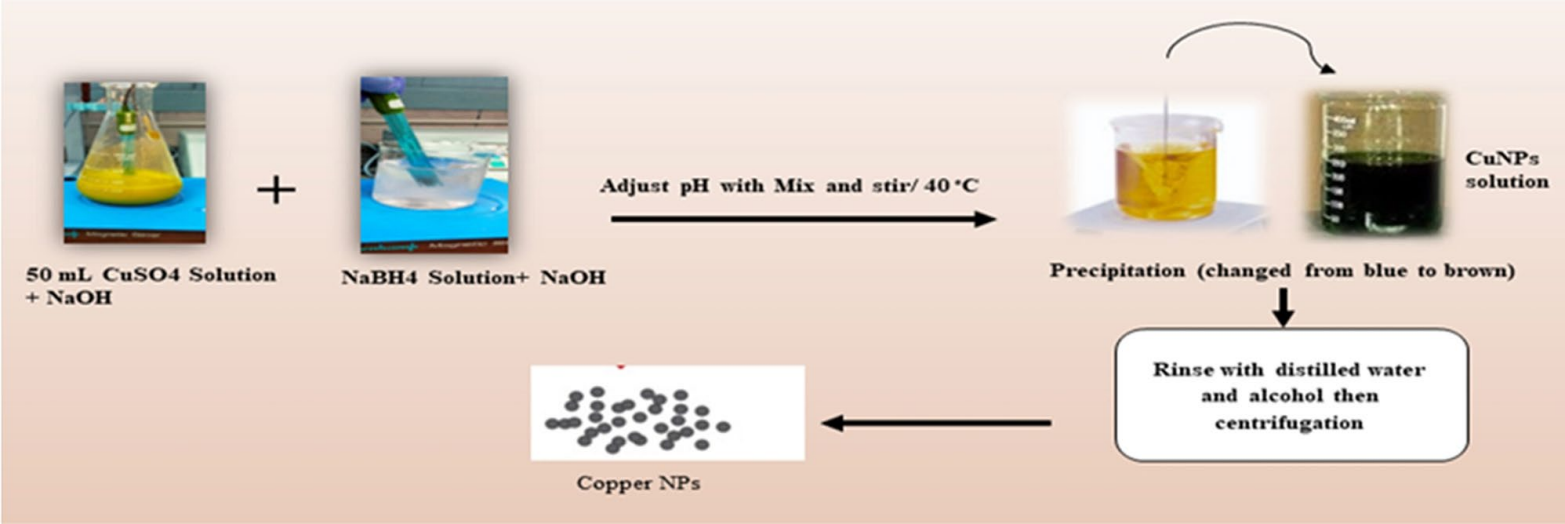
Scheme of copper nanoparticle preparation

The chemical synthesis of copper nanoparticles according to [41] was prepared using H_2_SO_4_ or NaOH solutions to adjust pH to the same value. The CuSO_4_ solution was then dispersed with 1% gelatin (mass fraction). The CuSO_4_ solution was then added drop by drop to the NaBH_4_ solution, which was stirred in a beaker at 313 K with a magnetic rod. The original blue color of the mixture turned brown as the Cu nanoparticles precipitated, as shown in Fig. 1.

### Characterization of copper nanoparticles

At the Regional Centre for Mycology and Biotechnology (RCMB), the generated CuNPs in aqueous solution were analyzed using the morphology image and size distribution graph using transmission electron microscopy (TEM) as follows: CuNPs were added to a drop that was applied to carbon-coated copper grids (CCG) and subjected to infrared light for 30 min. The JEOL-JEM 1010 transmission electron microscope analyzed the micrograph at 70 kV in the RCMB, Al-Azhar University. The X-ray diffraction (XRD-Model-D8 advance, BRUKER Germany) was used to evaluate the size and purity of the copper nanoparticles. The wide-angle X-ray diffraction spectrum of Cu-Ka radiation (λ = 0.1542 nm) The range of the spectrum is 4° to 70° in a step size of 0.02° (2θ) at 40 kV and 40 mA.

### Experimental design

Blue-green alga *Spirulina* sp. extract was obtained from the Unit of Algal Biotechnology, National Research Centre, Dokki, Egypt.The plants were sprayed with *Spirulina* sp. extract, which was added to distilled water to prepare the concentrations. In addition to the treatments with CuNPs and control plants (only treated with distilled water), there were a total of 13 treatments in this study. The seedlings were sprayed with treatments: CuNPs at three concentrations (500, 1000, and 1500 mg/L), and the interaction was as follows: (500 mg/L CuNPs + 0.5 g/L spirulina), (500 mg/L CuNPs + 1 g/L spirulina), (500 mg/L CuNPs + 1.5 g/L spirulina), (1000 mg/L CuNPs + 0.5 g/L spirulina), (1000 mg/L CuNPs + 1 g/L spirulina), (1500 mg/L CuNPs + 0.5 g/L spirulina), (1500 mg/L CuNPs + 1 g/L spirulina), and (1500 mg/L CuNPs + 1.5 g/L spirulina).

### Pot experiment

French basil seedlings (*Ocimum basilicum* L var. Grand Vert) have been obtained from the El-Kanater El-Khairiya farm in Kalyobia Governorate, which is associated with the Medicinal and Aromatic Plants Department of the Horticulture Research Institute. In both seasons, seeds were sown in the nursery in 14th -16th February at (11- 24^o^C and 50% humidity). After that, seedlings were planted in 1st April at (17- 29^o^C and 43% humidity). In our research, we used 30 cm-diameter pottery pots. Each pot was filled with 10 kg of a soil mixture (Table 1). The first half of the nitrogen and potassium fertilizer was applied 45 days after transplanting, and the second half was applied 45 days later. Every pot included two seedlings and was put in the complete sun under natural conditions in the Giza governorate. Plants were irrigated to field capacity with tap water for about 3 weeks while waiting for the development of basil plants. Throughout the growth phases and agricultural seasons of basil, the first foliar spraying was when the plant reached to the height 10–15 cm and the second spraying repeated after 10 days. Sprays were applied to every side of the leaves and stems with an atomizer sprayer, covering all above-ground parts of the basil plants. For better plant absorption of solutions, we must apply foliar spraying before sunrise, when the plant’s stomata are open and there is no wind or rain. The plants were irrigated regularly during the two seasons, in addition to other agricultural processes as usual. At the blooming stage, every measurement had been taken in May at (20- 32^o^C and 48% humidity).

**Table 1:** The physical and chemical characteristics of the soil

Clay%	Silt%	Fine sand%	Coarse sand%	Soil texture	pH	*N*	*P*_2_O_5_	K_2_O	Zn	Fe	B	Mn	Cu
									(ppm)				
39.23	24.34	27.13	2.24	Clay sand	7.33	22.41	103.9	154	3.29	1.94	2.29	0.47	0.46

### Vegetative growth characteristics

Plant fresh, dry weights (g), plant height, and number of branches were recorded at the stage of 50% flowering in the two seasons for each cut.

### Biochemical analysis

#### Determination of essential oil % and yield

According to the methods of [42] for determining the essential oil percentage, samples of fresh leaves (100 g) were subjected to hydro-distillation using a Clevenger apparatus. The distillation duration was 3 h for all samples. Also, oil yield (ml) per plant was calculated by multiplying the oil percentage by the fresh weight.

### Determination of photosynthetic pigments

According to [43], chlorophyll (a, b) and total carotenoids (mg/g fresh weight) were determined in fresh basil leaf samples. Fresh leaf samples of 0.2 g were homogenized in acetone (85% v/v) containing trace amounts of silica quartz and Na_2_CO_3_. Following that, the samples were passed via a glass funnel (G4) in the center. The remaining substance was washed several times with acetone until the filtrate became colorless. To the specified volume (25 ml), the whole extract was diluted with 85% acetone. At wavelengths of 660, 640, and 440 nm, the pigments have been compared to a blank of pure 85% acetone using a spectrophotometer. The following formulas were used to calculate the contents of the various leaf pigments:

Chlorophyll a (mg/L) = 9.784 E 660–0.99 E 640.

Chlorophyll b (mg/L) = 21.426 E 640–4.65 E 660.

Carotenoids (mg/L) = 4.695 E 440–0.268 (a + b).

### Chemical analysis of the essential oil using gas chromatography–mass spectrometry

The hydro-distilled essential oils were analyzed by gas chromatography-mass spectrometry (GC-MS) at the National Institute of Standard and Technology, NIST. using agarose chromatography (Hewlett-Packard model 5890), joined to a mass spectrometer (Hewlett-Packard-MS model 5970), and equipped with a DB5 fused silica capillary column (60 m, 0.32 mm i.d., 0.25 mm film thickness). The oven’s temperature was first kept at 50 °C for 5 min and then programmed from 50 to 250 °C at a rate of 4 °C/min. As the carrier gas, helium was employed at a flow rate of 1.1 mL/min. Diethyl ether (30 mL essential oil/mL diethyl ether) was used to dissolve the essential oil, and 2 mL of this solution was then injected into the GC with a split ratio of 1:10. The injection temperature was 220 °C. At 70 eV, electron impact mode (EI) mass spectra were produced, with a scan m/z range of 39 to 400 amu. By comparing the data from the NIST library of mass spectra with those of real compounds and published standards, isolated peaks were found [44]. The calculation of percentage composition of each oil was computed by the normalization method, which calculates the GC peak area by averaging three injections.

### Determination of lipid peroxidation

Malondialdehyde (MDA) contents were determined by the method designed by [45, 46]. Read the absorbance of the sample against the blank and standard against distilled water at 534 nm.

### Determination of enzymatic activity

For the reaction mixture extraction as described by [47], the fresh basil leaf sample was homogenized in 5 to 10 ml of cold buffer (50 mM potassium phosphate, pH 7.4, 1 mM EDTA, and 1 mL/L Triton X-100) per gram of tissue. After that, the mixture extraction was centrifuged for 15 min at 4,000 rpm at 4 °C. The supernatant was removed for assay and kept on ice.

### Catalase activity

Catalase (CAT) reacts with a known quantity of H_2_O_2_ by the [47] approach. The remaining hydrogen peroxide (H_2_O_2_) combines with 4-aminophenazone (AAP) and 3,5-dichloro-2-hydroxybenzene sulfonic acid (DHBS) to create a chromophore with a color intensity that is inversely proportional to the quantity of catalase in the initial sample. With a catalase inhibitor, the reaction is stopped after exactly one minute. At 510 nm, compare the sample to the sample blank and the standard to the standard blank.

### Glutathione peroxidase activity

The glutathione peroxidase (GPx) activity was assayed by a description of [48]. The enzyme reaction is started by adding the substrate (H_2_O_2_) and measuring the reaction’s absorbance at 340 nm. Then note the decline in absorbance at 340 nm/min over three minutes in comparison to deionized water. (GPx) activity in the sample is directly proportional to the rate of decline in the absorbance at 340 nm.

### Superoxide dismutase activity

SOD analysis depends on the enzyme’s capacity to prevent the phenazine methosulphate-mediated reduction of nitro blue tetrazolium dye, according to the assay created by [49]. For both the control and the sample, record the increase in absorbance at 560 nm for 5 min at 25 °C.

### Statistical analysis

This study used a completely randomized experimental design with three replicates. Applying the statistical analysis system (SAS) [50], we compared the one-way analysis of variance methods and treatment methods utilizing the LSD technique at *P* ≤ 0.05 of probability.

## Results and discussion

### Characterization of Cu-nanoparticles synthesized

**Fig. 2:**
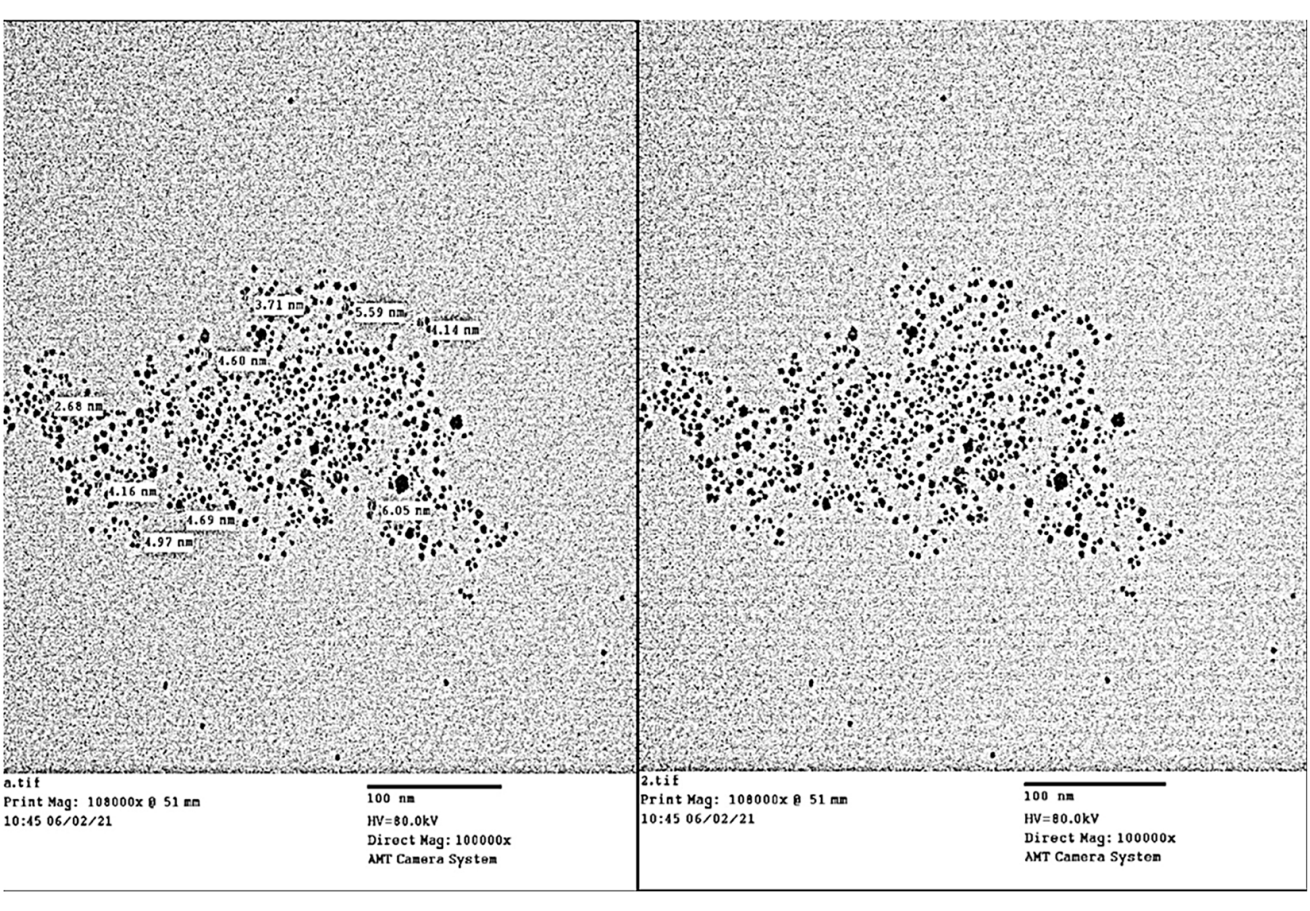
Transmission Electron Microscope (TEM) image of spherical copper nano particles

TEM analysis demonstrated the size and shape of synthesized CuNPs, as shown in Fig. 2. From the images, it is obvious that the morphology of CuNPs is almost spherical, having a size of less than 10 nm. Nanoparticles exhibit better physical properties if they are produced in small sizes, as the good fertilizer properties of copper nanoparticles are size-dependent. The micrograph in Fig. 2 showed nearly spherical nanoparticles with a meandiameter of 4.51 nm. The mean diameter wastaken from 9 nanoparticles; the minimum diameter was 2.68 nm, the maximum diameterwas 6.05 nm, and Dev(rms) = 0.945 nm.

**Fig. 3:**
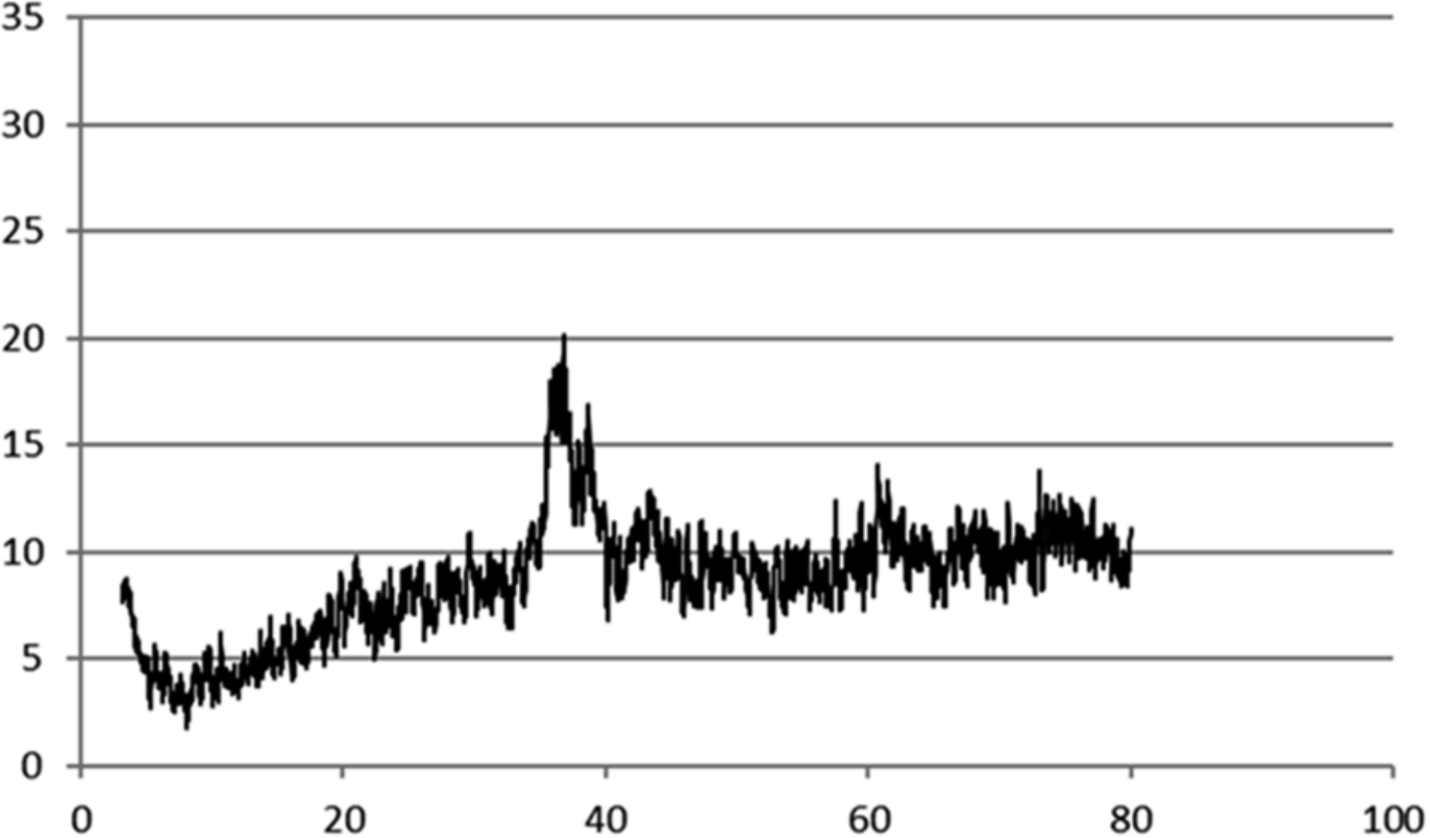
X-Ray diffraction plot

The sample was examined using an X-ray powder diffractometer (XRD) to determine the structure and crystalline nature of the CuNPs. When the XRD spectrum data were compared to standards, it was discovered that the CuNPs generated peaks at 2 µ values, which correspond to 1.52667, 2.08955, 2.32762, and 2.44519 of 44,519 Bragg reflections for copper metal (Fig. 3).

The pattern exhibited four intense peaks in the spectrum of 2 theta: 36.725, 38.652, 43.264, and 60.605. From the XRD results, the crystallite size was synthesized with an average size of 4.2 nm. A face-centered cubic phase of crystalline copper was indicated by the presence of Bragg reflections that matched card number 174,091 from the ICSD card, which corresponds to the (0 1 1), (1 1 1), and (0 0 2) planes, these findings were in agreement with [51, 52].

**Table 2:** Fresh and dry weights of French basil plants, after the treatments foliar spraying

Treatment	Fresh weight(g)		Dry weight(g)	
	Season(1)	Season(2)	Season(1)	Season(2)
Control	71.45 ± 0.5 ^i^	92.77 ± 0.77^h^	17.91 ± 1.4^h^	15.67 ± 0.7^cd^
500 mg/L CuNPs	85.12 ± 1.1^e^	102.69 ± 0.69^e^	22.94 ± 1.0^ef^	17.09 ± 1.0^c^
1000 mg/L CuNPs	80.61 ± 0.3^f^	88.02 ± 1.0^i^	21.45 ± 1.2^fg^	14.44 ± 1.2^d^
1500 mg/L CuNPs	73.99 ± 0.1^h^	64.51 ± 0.8^k^	20.73 ± 1.0^g^	14.34 ± 0.9^d^
500 mg/L CuNPs + 0.5 g/L spirulina	93.41 ± 0.4^d^	115.69 ± 1.0^d^	25.86 ± 1.1^bc^	21.95 ± 1.0^b^
1000 mg/L CuNPs+ 0.5 g/L spirulina	86.08 ± 1.0^e^	100.5 ± 1.5^fg^	23.32 ± 1.1^de^	14.58 ± 1.3^d^
1500 mg/L CuNPs+ 0.5 g/L spirulina	74.29 ± 0.3^h^	85.97 ± 1.0^j^	21.89 ± 1.1^fg^	16.7 ± 1.1^c^
500 mg/L CuNPs + 1 g/L spirulina	95.85 ± 0.9^c^	136.69 ± 1.0^b^	27.17 ± 1.0^ab^	21.98 ± 1.0^b^
1000 mg/L CuNPs + 1 g/L spirulina	92.19 ± 1.0^d^	123.56 ± 1.0^c^	24.16 ± 1.0^cde^	20.38 ± 1.1^b^
1500 mg/L CuNPs + 1 g/L spirulina	76.697 ± 0.1^g^	99.06 ± 1.0^g^	23.15 ± 1.0^def^	17.2 ± 1.1^c^
500 mg/L CuNPs + 1.5 g/L spirulina	122.26 ± 1.0^a^	169.71 ± 1.1^a^	27.87 ± 1.9^ab^	24.0 ± 1.0^a^
1000 mg/L CuNPs + 1.5 g/L spirulina	101.66 ± 1.2^b^	102.1 ± 1.0^ef^	25.09 ± 1.0^cd^	21.23 ± 1.1^b^
1500 mg/L CuNPs + 1.5 g/L spirulina	92.94 ± 1.0^d^	100.1 ± 1.0^g^	24.23 ± 1.1^cde^	20.87 ± 0.9^b^
LSD	1.398793343	1.678332759	2.000540928	1.74274742

Data are expressed as mean values ± standard deviation; LSD refers to the least significant difference test. In each column, the same letter means non-significant difference, while different letters mean significant difference at *p* ≤ 0.05

According to the results in Table 2, the fresh and dry weight increased with 500 mg/L Cu NPs achieving the highest fresh and dry weight as follows 85.12, 102.687 g and 22.94, 17.09 g at the two seasons, respectively, then decreased with increasing CuNPs concentration. Along the same line [53], confirmed that spraying CuNPs at 50, 100, and 200 mg/L on pepper seedlings increased the shoot biomass, while treatments at 200 mg/L recorded the highest biomass. Furthermore, the superinfluence of CuNPs on the growth of pigeon pea (*Cajanuscajan* L.) seedlings was significant, whereas treatment with 20 ppm CuNPs presented a highly significant increase in fresh and dry biomass compared with untreated plants [54]. Additionally [55], indicated that the application of CuO nanoparticles significantly improved the biomass of wheat plants. Therefore [56], explained that CuO nanoparticles increased ion release and greater bioavailability due to the size-dependent physiochemical characteristics of soil nanoparticles over bulk particles, making them more capable of maximizing crop output [57]. As indicated in Table 1, the interaction between the foliar spray of CuNPs and spirulina extract was more effective than the treatment of CuNPs individually. Therefore, foliar spray of 500 mg/L of CuNPS + 1.5 g/L algae extract was the best treatment on fresh and dry weight as follows 122.257, 169.713 g, and 27.87, 24.04gat the two seasons, respectively. Our results are in harmony with [32], who recorded that the combination of 2000 ppm CuNPs with 4000 ppm ZnNPs achieved the best fresh and dry weight of basil roots, while the minimum dry root weight was noted in the plants treated with distilled water, which was followed by 4000 ppm CuNPs without ZnNPs. However [58], found that increasing spirulina algae from 25:100 when combined with nitrogen fertilization at 80 and 100 kg/fed improved considerably wheat growth and yield.

**Table 3:** Plant height and the number of branches of French basil plantafter the treatments foliar spraying in two seasons

Treatment	Plant height(cm)		Number of branches /plant	
	Season (1)	Season(2)	Season(1)	Season(2)
Control	41.40 ± 0.4^f^	35.80 ± 0.4^h^	5.00 ± 1.00^c^	5.76 ± 1.00^bc^
500 mg/L CuNPs	48.07 ± 1.0b^c^	47.93 ± 1.0^de^	7.00 ± 1.00^ab^	6.00 ± 1.00^bc^
1000 mg/L CuNPs	46.53 ± 0.01^de^	44.47 ± 0.001^f^	5.00 ± 1.00^c^	5.00 ± 1.00^c^
1500 mg/L CuNPs	45.67 ± 1.0^e^	43.30 ± 1.00^g^	5.00 ± 1.00^c^	5.00 ± 1.00^c^
500 mg/L CuNPs + 0.5 g/L spirulina	48.73 ± 1.0^bc^	48.60 ± 1.00^cd^	8.00 ± 1.00^a^	6.00 ± 1.00^bc^
1000 mg/L CuNPs+ 0.5 g/L spirulina	46.33 ± 1.1^de^	48.17 ± 0.001^cde^	7.00 ± 1.00a^b^	6.00 ± 1.00^bc^
1500 mg/L CuNPs+ 0.5 g/L spirulina	46.30 ± 1.0^de^	43.43 ± 0.01^fg^	6.00 ± 1.00^bc^	6.00 ± 1.00^bc^
500 mg/L CuNPs + 1 g/L spirulina	51.03 ± 0.01^a^	49.07 ± 0.01^c^	8.00 ± 1.00^a^	7.00 ± 1.00^ab^
1000 mg/L CuNPs + 1 g/L spirulina	49.26 ± 1.1^b^	48.60 ± 1.4^cd^	8.00 ± 1.00^a^	7.00 ± 1.00a^b^
1500 mg/L CuNPs + 1 g/L spirulina	47.53 ± 1.0^cd^	47.27 ± 0.001^e^	7.00 ± 1.00^ab^	6.00 ± 1.00^bc^
500 mg/L CuNPs + 1.5 g/L spirulina	51.57 ± 0.01^a^	51.90 ± 0.1^a^	9.00 ± 1.5^a^	8.00 ± 1.00^a^
1000 mg/L CuNPs + 1.5 g/L spirulina	51.50 ± 0.1^a^	50.17 ± 0.005^b^	9.00 ± 1.5^a^	7.00 ± 1.00^ab^
1500 mg/L CuNPs + 1.5 g/L spirulina	47.97 ± 0.01^c^	49.20 ± 0.2^bc^	8.00 ± 1^ab^	7.00 ± 1.00^ab^
LSD	1.290086696	1.290086696	1.762300627	1.678332759

Data are expressed as mean values ± standard deviation; LSD refers to the least significant difference test. In each column, the same letter means non-significant difference, while different letters mean significant difference at *p* ≤ 0.05

Concerning our results in Table 3, we found that the highest plant height (48.067 cm and 47.933 cm) and number of branches (7 and 6) were achieved by spraying 500 mg/L of CuNPs, in the two seasons, respectively, followed by spraying 1000 mg/L of CuNPs, while the least plant height and number of branches resulted from untreated plants followed by 1500 ppm of CuNPs. It is likely that [32] noticed that a better leaf number was achieved by spraying basil plants with 1000 ppm CuNPs than with 2000 and 4000 ppm CuNPs. Many researchers have shown that foliar spraying chili plants with CuNPs improved leaf number, pod number, pod length, and plant heightas well as [34, 54], and found that the maximum shoot and root length of the pigeon pea plant occurred with CuNPs application. Additionally, foliar fertilizers aid in preventing toxicity symptoms that could develop after applying the same microelements to the soil [20]. In this research we found that plant height and the number of leaves were increased with the combination of CuNPs with biofertilizer (spirulina) at various concentrations therefore, the spraying of 500 mg/L of CuNPs + 1.5 g/L algae extract produced significantly best plant height and a number of branches in both seasons as follows (51.567, 51.900 cm/plant) (9.00, 10.00 /plant), respectively.These findings were in line with those of [59], who proved that the effectiveness of the combined treatment of 200 mM NaCl with 100 mg/L of *Spirulina platensis* enhanced growth with increasing photosynthetic pigment content and plant yield, which is due to reduced ROS-induced oxidative damage and decreased the damage to DNA in salt-stressed *Phaseolus vulgaris* compared with treatments of salt stress without spirulina. Similarly [60], who found the application of 192 kg of fertilizer N/ha combined with destructed spirulina at 6, 12, or 18 g cells/ha on cotton, recorded the highest number of open bolls and, additionally, the best plant height and fiber length. Furthermore [61], obtained similar trends by combining spirulina and organic manure in a 50:50 ratio with the *Phaseolusaureus* plant, which significantly improved shoot growth and yield.

### Chemical analysis

#### Essential oil percentage (%) and yield

In terms of oil percentage, we found that CuNPs had a stimulatory effect on oil yield generally since CuNPs at 500 mg/L recorded the best of other CuNP concentrations on essential oil (%) and essential oil yield (ml), which recorded 0.120, 0.100% and 8.593, 10.9531 ml/plant in both seasons, respectively (Table 4), and the yield decreased with increasing concentration of CuNPs. Additionally, non-sprayed plants had the lowest value followed by 1500 mg/LCuNPs, then 1000 mg/L CuNPs. This was a result of stimulation of fresh material or plants, photosynthetic activity, and essential oil products.

**Table 4:** The essential oil percentage (%) and yield ml/plant of the French basil plant after treatment foliar application in two seasons

Treatment	Oil %		Oil yield ml /plant	
	Season (1)	Season (2)	Season (1)	Season(2)
Control	0.055 ± 0.001^h^	0.067 ± 0.011^g^	4.202 ± 0.10^l^	6.164 ± 0.111^k^
500 mg/L CuNPs	0.120 ± 0.010^d^	0.100 ± 0.001^f^	8.593 ± 0.10^i^	10.953 ± 0.011^g^
1000 mg/L CuNPs	0.100 ± 0.001^efg^	0.067 ± 0.001^g^	8.394 ± 0.06^j^	9.027 ± 0.011^i^
1500 mg/L CuNPs	0.093 ± 0.001^g^	0.060 ± 0.001^g^	8.031 ± 0.01^k^	5.929 ± 0.010^l^
500 mg/L CuNPs + 0.5 g/L spirulina	0.120 ± 0.010^d^	0.109 ± 0.001^def^	11.528 ± 0.11^e^	12.397 ± 0.101^c^
1000 mg/L CuNPs+ 0.5 g/L spirulina	0.093 ± 0.001^g^	0.105 ± 0.001^ef^	10.114 ± 0.06^h^	11.377 ± 0.11^f^
1500 mg/L CuNPs+ 0.5 g/L spirulina	0.110 ± 0.010^def^	0.100 ± 0.002^f^	8.403 ± 0.10^j^	6.857 ± 0.111^k^
500 mg/L CuNPs + 1 g/L spirulina	0.173 ± 0.011^b^	0.123 ± 0.001^c^	15.071 ± 0.01^b^	17.595 ± 0.101^c^
1000 mg/L CuNPs + 1 g/L spirulina	0.099 ± 0.001^fg^	0.120 ± 0.010^cd^	10.761 ± 0.11^f^	13.114 ± 0.001^d^
1500 mg/L CuNPs + 1 g/L spirulina	0.113 ± 0.011^de^	0.113 ± 0.011^cde^	10.482 ± 0.11^g^	10.543 ± 0.111^h^
500 mg/L CuNPs + 1.5 g/L spirulina	0.380 ± 0.010^a^	0.163 ± 0.011^a^	21.174 ± 0.11^a^	23.689 ± 0.110^a^
1000 mg/L CuNPs + 1.5 g/L spirulina	0.173 ± 0.011^b^	0.153 ± 0.011^a^	13.209 ± 0.10^c^	18.862 ± 0.111^b^
1500 mg/L CuNPs + 1.5 g/L spirulina	0.133 ± 0.001^c^	0.140 ± 0.010^b^	12.317 ± 0.001^d^	13.174 ± 0.001^d^
LSD	0.012902719	0.012250915	0.173442611	0.14305719

Data are expressed as mean values ± standard deviation; LSD refers to the least significant difference test. In each column, the same letter means non-significant difference, while different letters mean significant difference at *p* ≤ 0.05

Similar to our study [35], applied CuNPs as a foliar spray at 0.5, 1.0, and 1.5 g/L, which improved essential oil percentages more than untreated plants, and recorded that 1.5 g/LCuNPs achieved the highest value of oil yield and essential oil percentage of peppermint.

The results indicated that the interaction of CuNPs and Spirulina extract was better at improving oil percentage and oil yield than the individual treatments. 500 mg/L Cu NPs with 1.5 g/L presented a remarkable increase in oil% and yield. Application of 0.1% spirulina extract combined with Cd and Pb on rosemary plants, increasing oil% and yield better than spirulina with Cd, which enhanced oil production compared with control [62]. On the other hand [28], used a combination of 10% spirulina extract and 6.72 mL of compost tea; additionally, 50 and 75% nitrogen fertilizer were applied to fennel plants, resulting in no differences in the percentage of essential oils produced from treated and untreated plants.In addition, spirulina is used as a great natural biofertilizer due to its ability to supply high amounts of bioactive compounds (polysaccharides, amino acids, phytohormones, etc.), which improve the growth of plants and their capacity to adapt to biotic and abiotic stresses [63].

### Photosynthetic pigments analysis

Our work proved that when CuNPs were applied at any concentration (500, 1000, and 1500 mg/L), they stimulated photosynthetic pigments better than control. As shown in Table 5, we found that 500 mg/L resulted in the highest content of chlorophyll “a”, chlorophyll “b,” and carotenoids in the two seasons, respectively. The second enhancing treatment was 1000 mg/L, followed by 1500 mg/L. CuNPs have a stimulatory effect on growth. This is due to the ability of CuNPs to play an important role in photosynthesis, improve phosphorylation, and transport electrons through the light reaction to automatically increase enzyme activity in the dark phase and initiate carbon and nitrogen metabolism [64].

**Table 5:** Chlorophyll (a, b) and carotenoids content in fresh leaves of French basil plants after foliar application in two seasons

Treatment	Clorophyl (A)		Clorophyl (B)		Carotenoids	
	Season (1)	Season(2)	Season(1)	Season(2)	Season(1)	Season(2)
Control	0.476 ± 0.001^i^	0.536 ± 0.001^g^	0.224 ± 0.01^k^	0.251 ± 0.01^j^	0.334 ± 0.01^h^	0.362 ± 0.001^g^
500 mg/LCuNPs	0.809 ± 0.001^e^	0.841 ± 0.011^cd^	0.317 ± 0.001^f^	0.331 ± 0.001^g^	0.389 ± 0.001^d^	0.394 ± 0.001^d^
1000 mg/L CuNPs	0.747 ± 0.001^g^	0.742 ± 0.001^e^	0.306 ± 0.001^g^	0.310 ± 0.01^h^	0.374 ± 0.001^e^	0.384 ± 0.01^e^
1500 mg/L CuNPs	0.688 ± 0.001^h^	0.637 ± 0.001^f^	0.263 ± 0.001^j^	0.275 ± 0.001^i^	0.354 ± 0.001^g^	0.372 ± 0.001^f^
500 mg/L CuNPs + 0.5 g/L spirulina	0.860 ± 0.01^b^	0.843 ± 0.001^cd^	0.345 ± 0.001^c^	0.362 ± 0.001^e^	0.409 ± 0.001^b^	0.419 ± 0.001^b^
1000 mg/L CuNPs+ 0.5 g/L spirulina	0.799 ± 0.001^f^	0.744 ± 0.001^e^	0.320 ± 0.001^ef^	0.341 ± 0.001^f^	0.386 ± 0.001^d^	0.397 ± 0.001^d^
1500 mg/L CuNPs+ 0.5 g/L spirulina	0.747 ± 0.001^g^	0.739 ± 0.001^e^	0.276 ± 0.001^i^	0.281 ± 0.001^i^	0.364 ± 0.001^f^	0.382 ± 0.001^e^
500 mg/L CuNPs + 1 g/L spirulina	0.817 ± 0.001^df^	0.847 ± 0.001^bc^	0.358 ± 0.001^b^	0.395 ± 0.001^ab^	0.422 ± 0.005^a^	0.423 ± 0.001^b^
1000 mg/L CuNPs + 1 g/L spirulina	0.815 ± 0.001^df^	0.853 ± 0.001^ab^	0.325 ± 0.001^d^	0.384 ± 0.001^c^	0.390 ± 0.001^d^	0.408 ± 0.001^c^
1500 mg/L CuNPs+ 1 g/L spirulina	0.810 ± 0.001^e^	0.747 ± 0.01^e^	0.301 ± 0.001^g^	0.340 ± 0.001^f^	0.378 ± 0.001^e^	0.398 ± 0.001^d^
500 mg/L CuNPs + 1.5 g/L spirulina	0.90 ± 0.001^a^	0.856 ± 0.01^a^	0.374 ± 0.001^a^	0.399 ± 0.001^a^	0.428 ± 0.001^a^	0.432 ± 0.001^a^
1000 mg/L CuNPs + 1.5 g/L spirulina	0.847 ± 0.001^g^	0.836 ± 0.001^d^	0.348 ± 0.001^c^	0.389 ± 0.001^bc^	0.400 ± 0.001^d^	0.421 ± 0.001^b^
1500 mg/L CuNPs + 1.5 g/L spirulina	0.818 ± 0.001^d^	0.747 ± 0.01^e^	0.322 ± 0.001^de^	0.371 ± 0.001^d^	0.386 ± 0.001^d^	0.411 ± 0.001^c^
LSD	0.008066928	0.008468771	0.008066928	0.008468771	0.00728105	0.004926238

Data are expressed as mean values ± standard deviation, LSD refers to least significant difference test, in each column the same letter means non-significant difference, while different letters mean significant difference at *p* ≤ 0.05

Our results were confirmed by [32], who treated basil plants with 0 ppm ZnNPs and 2000 ppm CuNPs, which resulted in the maximum chlorophyll content (0.38 mg/g FW). Corn plant leaves showed a positive response to Cu treatments in terms of chlorophyll concentration [65]. Along with [66], copper spraying had a favorable impact on the chlorophyll content of sub-tropical peaches, which is consistent with the findings of the current study. On the other hand [67], indicated that nanoparticles, especially metals, can accumulate chlorophyll content through photosynthesis and chlorophyll content. Regarding the reproductive stage, the application of copper nanoparticles helps to promote flowering in plants, which stimulates fruiting per plant [68]. Using 30 ppm CuNPs with soil produced greatly developed the chlorophyll content of the wheat plants. In our work, in general, the maximum accumulation of basil photosynthetic pigments was achieved with the combined treatments of biofertilizer (Spirulina) and nanofertilizer (CuNPs) compared to each treatment alone. Thus, 500 mg/L CuNPs + 1.5 g/L spirulina extract resulted in the highest content of chlorophyll “a”, chlorophyll “b,” and carotenoids during two seasons, respectively. Followed by 1000 mg/L CuNPs + 1.5 g/L spirulina extract; moreover, 1500 mg/L CuNPs + 1.5 g/L spirulina resulted in the least content. Our results match those obtained in previous studies that sprayed basil plants with the combined treatment of CuNPs with Zn NPs and found a high increase in chlorophyll a, chlorophyll b, total chlorophyll, and carotenoids contents in the leaves [69]. Previous research proved that biofertilizers like *Spirulina platensis* have biological functions in the detoxification of some plant organic compounds and the absorption of heavy metals [70–72]. At the same line [62], achieved a great improvement in photosynthetic pigments (mg/g dry weight leaves) in rosemary plants by treating plants with *Spirulina platensis* as foliar application combined with heavy metal. In addition, the interaction treatment was better than the heavy metal individual. Furthermore [73], applied a good combination of 50% N (inorganic N) with 50% plant compost supplemented with *Spirulina platensis* at 10 mL on sweet grapevines, resulting in significantly enhanced growth features, yield, berry cluster weight, and quality. Similarly [60], reported that foliar spray, the interaction between N fertilizer application and Spirulina (destructed cells), accumulated the maximum content of photosynthetic pigments in cotton plants. Previous research proved that using an extract of *Spirulina platensis* as a foliar spray with plants led to super enhancement due to Spirulina being rich in plant hormones such as auxins, cytokinins, gibberellins, free amino acids, and nutritive compounds, which are growth promoters for improving plant growth and yield [74–76]. Therefore [77], reported the chlorophyll “a”, chlorophyll “b,” and carotenoids contents of the *Faba* L. plant improved considerably under salt stress after applying spirulina extract to the plant to overcome salt stress.

#### Chemical analysis of the essential oil

In our work, GC–MS via chemometric techniques for analysis of the volatile components of French basil (*Ocimum basilicum* L var. Grand Vert) plants with foliar spray application of CuNPs and spirulina as shown in Table 6. In our leaves essential oil samples, there were approximately 18 components identified for every treatment.

**Table 6:** The influences of spraying a combination of 500 mg/LCuNPs + 1.5 g/L spirulinaon the composition of essential oil French basil leaves in the first year of the experiment

(%)Components	Control	500 mg/L CuNPs	500 mg/LCuNPs + 1.5 g/L spirulina
Linalool	30.33	28.71	38.19
Eugenol	11.59	18.94	15.63
Methyl chavicol	1.31	1.41	1.45
Methyl Eugenol	2.19	3.05	2.16
Geranyl propanoate	0.94	1.4	1.03
Bornyl acetate	1.37	1.29	1.3
Carvacrol	0.42	---	----
α-Cubebene	0.5	0.67	0.56
β –Elemene	4.58	----	----
α-trans-Bergamotene	4.06	4.47	6.32
β-Farnesene	0.45	0.59	0.56
γ-Muurolene	0.57	0.78	0.7
α-selinene	2.56	3.55	2.85
β-Bisabolene	1.76	2.87	1.77
γ-Cadinene	0.62	0.31	0.71
δ-Cadinene	3.03	4.3	3.61
cis-Nerolidol	0.55	0.88	0.46
γ-Eudesmol	1.05	1.47	1.11

According to our study, we found that the major compounds of basil essential oil were linalool, methyl chavicol, eugenol, methyl eugenol, and α-trans-Bergamotene. Using foliar applications of biofertilizer and nanofertilizer caused a positive effect on the biosynthesis of highly valuable compounds in essential oils. Linalool recorded a maximum value of 38.19%, which was obtained with the combined treatment (500 mg/LCuNPs + 1.5 g/L spirulina), followed by control and 500 mg/L CuNPs. Similarly [78], produced the maximum level of the main component Linalool by treating the *Ocimum basilicum* L. plant with 6 g/L dry yeast and 0 g/L seaweed.In addition, the greatest content of eugenol andmethyl chavicol was obtained from plants treated with 500 mg/LCuNPs, followed by 500 mg/L CuNPs + 1.5 g/L spirulina and control. Additionally, the percentage of methyl chavicol ranged from 1.31% in the untreated plants to 1.45% in plants treated with 500 mg/L nanocopper, whereas the highest content of methyl eugenol occurred with 500 mg/L CuNPs followed by control and 500 mg/L CuNPs + 1.5 g/L spirulina.

In our observation, the maximum content of linalool and methyl chavicol was found in plants treated with 500 mg/L CuNPs + 1.5 g/L spirulina, while the greatest amounts of eugenol and methyl eugenol were achieved in plants treated with 500 mg/L CuNPs. On the other hand, CuNps affected some contents negatively, such as carvacrol and β-elemene, compared to the control.Notably, Geranyl propanoate, α-Cubebene, β-Farnesene, γ-Muurolene,α-selinene, β-Bisabolene, γ-Cadinene, δ-Cadinene, cis-Nerolidol, and γ-Eudesmol increased significantly with 500 mg/L CuNPs, followed by 500 mg/L CuNPs + 1.5 g/L spirulina compared with control. At the same time, Bornyl acetate and α-trans-Bergamotene increased with 500 mg/L CUNPs + 1.5 g/L spirulina, followed by 500 mg/L CuNPs compared with control [79]. reported that dealing basil plants with organic foliar fertilizers led to an accumulation of oil content; additionally, it enhanced biosynthesis of the important compounds of essential oils; and it recorded the presence of linalool, methyl chavicol, eugenol, γ-cadinene, α-bergamotene, cubenol, and β-elemene as major compounds in every sample.Our findings were in contrast with [80], who referred to the essential oil of *O.basilicum* as having four main chemotypes (linalool, methyl chavicol, methyl eugenol, and methyl cinnamate), which have superoxide scavenging activity and DPPH reduction. Furthermore [81], proved that when using the NPK with active dry yeast with *Ocimum basilicum* L. plants, the identification of essential oil basil leaves showed that 19 components were detected at every treatment. Linalool was also one of the four main constituents. In addition, the combination of the NPK with active dry yeast with every treatment individual had a substantial impact on the primary chemical constituents of the *Ocimum basillicum* leaf essential oil. Moreover [82], studied the essential oils extracted from basil leaves using hydro-distillation and found the presence of linalool 48.4%, methyl chavicol 14.3%, methyl eugenol 3.7%, α-bergamotene 2.5%, eugenol 2.4%, 1,8-cineole 7.3%, (E)-Methylcinnamate 2.3%, and α-bisabolol 4.1%.

### Lipid peroxidation quantification

**Fig. 4:**
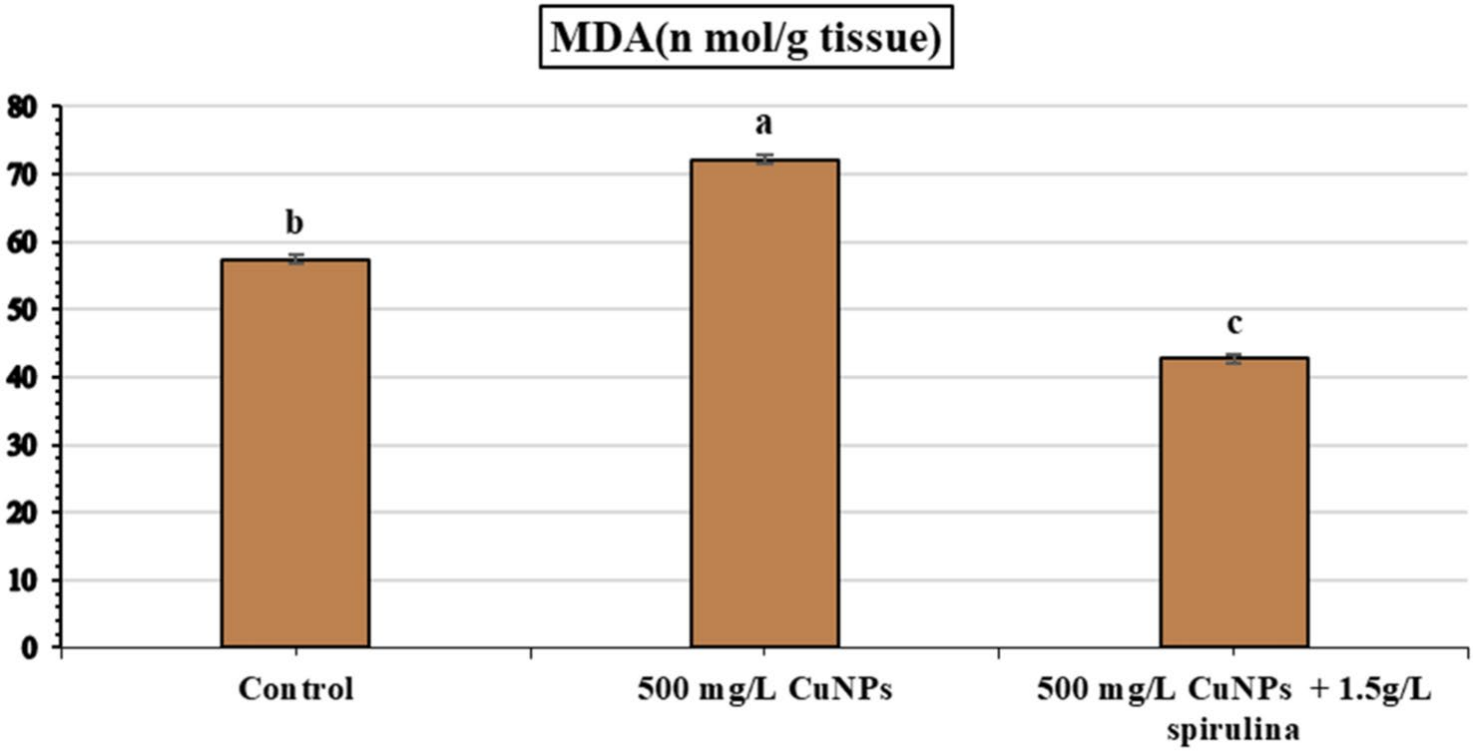
The influence offoilar application of 500 mg/LCu NPsand 500 mg/ LCuNPs+. 1.5 g/L spirulina extract on the content MDA compared with control in French basil plants. Vertical bars represent the means of three independent determination standard error (SE). The different letters are significantly different between treatments at the 0.05 level

Our results presented in Fig. 4 showed that MDA activity in fresh basil leaves recorded a significant increase (72.2 nmol/g tissue) with nano fertilizer application (500 mg/L CuNPs), though the control recorded 57.39 nmol/g tissue. On the other hand, spraying (500 mg/L CuNPs + 1.5 g/L spirulina) reduced the MDA level to 42.787 nmol/g tissue compared to 500 mg/L CuNPs.Previous research indicated that raising the contents of MDA and ROS activation via the application of CuO nanoparticles with various concentrations [83] Furthermore [84], reported that the application of CuNPs also produced copper ions on *Elodeadensa* plants, indicating an increase in lipid peroxidation MDA content. Also [56], explained that MDA contents and reactive oxygen species (ROS) are directly related to each other as they are considered indicators of slight oxidative stress via CuO nanoparticle treatments.

It is important to point out that nano-priming causes highly antioxidant activity and the production of secondary metabolites as a result of eliciting reactive oxygen species (ROS), which is the first signal for different biological reactions that occur for stress tolerance [85]. On the contrary, foliar application of biofertilizer *S. platensis* (100 mg/L) on *Phaseolus vulgaris* plants under salinity stress achieved decreasing DNA damage along with ROS-induced oxidative damage. Thus, the growth improvement of S. platensis is due to its bioactive compounds, plant growth regulators, which scavenge ROS; therefore, algal application has been used in recent sustainable agriculture to decrease stress hazards [59].

#### Enzymatic activity assays

**Fig. 5:**
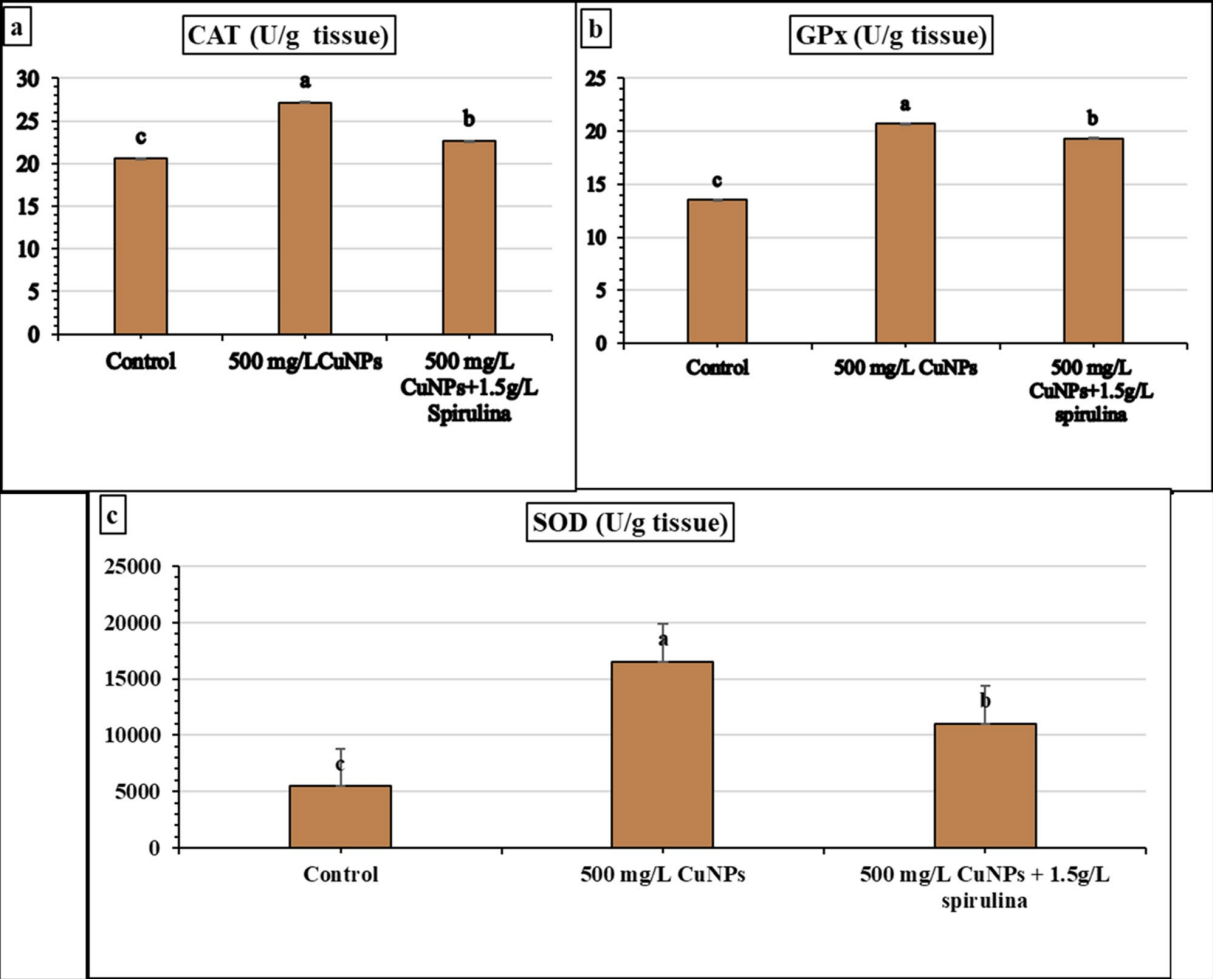
The influence of the application of 500 mg/LCuNPsand a combination of 500 mg/LCuNPs and 1.5 g/L spirulina extract on the enzyme activity of (**a**) CAT, (**b**) GPx, and (**c**) SOD (U/g tissue) in French basil plants. Vertical bars represent the means of three independent determination standard error (SE). The different letters are significantly different between treatments at the 0.05 level

Effects of nano fertilizer and combination with biofertilizer (spirulina) application related to induced resistance and basil plant defense enzymes were studied from the experiment on treated and untreated basil leaves. In this study, all treatments caused a significant change in the enzyme activity of CAT, GPx, and SOD. According to Fig. 5, given result, it was reported that the activity of CAT increased with the application of 500 mg/L CuNPs followed by 1.5 g/L spirulina extract + 500 mg/L CuNPs as follows 27.18, 22.65 U/g tissue, respectively, compared with the control, which recorded 20.59 U/g tissue, although CAT activity decreased with the application of spirulina extract at 1.5 g/L as follows (21.313 U/g tissue).Moreover, GPx activity increased with 500 mg/LCuNPs followed by 1.5 g/L spirulina extract + 500 mg/LCu NPs as follows 20.711, 19.342 U/g tissue, respectively, compared with control, whereas using 1.5 g/L spirulina extract decreased GPx activity as follows 17.57 U/g tissue. Additionally, SOD activity recorded the highest value with 500 mg/L CuNPs, followed by 1.5 g/L spirulina extract + 500 mg/L CuNPs as follows 16544.12, 11026.4 U/g tissue, and the least value observed with 1.5 g/L spirulina extract, which recorded 10029.4 U/g tissue compared with control.

Our results, in agreement with many research studies [32], proved that foliar application to basil plants with the interaction of 4000 ppm ZnNPs and 2000 ppm CuNPs had a positive impact on the antioxidant activity of the basil plants. Similarly [86], reported that CuNPs application with tomato plants achieved a positive impact on CAT and SOD activity, which played an important role in removing ROS and, in addition, enhancing bioactive constituents for high-quality fruits. Therefore [64], explained that CuNPs improved photosynthesis, light reactions, and the chain of electron transport, which induced the activity of enzymes. Also [87], revealed that Cu with a high concentration causes cellular damage as a result of the increasing activity of ROS and enzymes (CAT, SOD, APX).Conversely, with the application of spirulina with *Viciafaba* L. plants salt stressed, the enzymes sodium oxide dismutase and catalase recorded high values in plants with salinity alone and reduced enzyme activity when the combined treatment of spirulina with salt stress was used [77]. Along with this [88], indicated that using 5% of spirulina application had a positive effect on the growth and enzyme activity of the Eruca sativa plant. According to our result [89], affirmed that through activating the antioxidant defense system to reduce the negative effects of oxidative stress achieved with foliar application, the combined treatments of carbonate-precipitating bacteria (CCPB) and Si-NPs were superior to the separate treatments of CCPB or Si-NPs in enhancing physio-biochemical characteristics and enzymatic antioxidant activities. This increased tolerance and improved wheat plant (*Triticuma estivum* L.) production in sandy soils under semi-arid environmental conditions Hence, these methods, when paired with others, caused plant leaves to stay green, postponed senescence, and increased photosynthetic efficiency and chlorophyll content to maintain healthy plants. Therefore, these advancements in antioxidant defense mechanisms aid in limiting oxidative damage.

## Conclusion

According to the results of this work, applying foliar spraying of CuNPs in French basil suggests a stronger oxidative stress response on the plants than the treatment of the combination between spirulina extract and CuNPs in different concentrations, we found that the treatment of 500 mg/L CuNPs + 1.5 g/L spirulina extract had the best effect in increasing growth parameters, essential oil percentage, and pigments, when compared with control and other treatments. Also, these treatments showed significant differences in enzyme activities. Finally, our results approved that the combination of CuNPs and green algae could have a positive effect as a biofertilizer more than the individual application of nanoparticles. Therefore, the research on different plant materials for fertilization with other nano-particles and green synthesizers would be important for the future study.

## Data Availability

All data generated and analyzed during this study are included in this published article.

## Abbreviations

CuNPs: Copper Nanoparticles
SOD: Superoxide Dismutase
GPX: Glutathione Peroxidase
CAT: Catalase
MDA: Malondialdehyde
ROS: Reactive Oxygen Species
TEM: Transmission Electron Microscope
XRD: X-Ray Diffraction Method

## References

[1] -Marchioni I, Najar B, Ruffoni B, Copetta A, Pistelli L (2020). Bioactive compounds and aroma profile of some Lamiaceae flower. Plants.

[2] Simon JE, Morales MR, Phippen WB, Vieira RF, Hao Z. Basil: a source of aroma compounds and a popular culinary and ornamental herb. Perspectives on New Crops and New Uses. 1999; 499–505.

[3] -Sami MKH, Ali MA, Gaber MK, Sidky MMA, Farroh KY (2022). Growth, Productivity and Quality of Sweet basal in relation to Minerals, nanoparticles of Chitosan for NPK fertilization. J Biology Sci.

[4] ZagotoM, Cardia GFE, da Rocha EMT, Mourão KSM, Janeiro V, et al. Biological activities of basil essential oil: a review of the current evidence. Res Soc Dev. 2021;10(12). 10.33448/rsd-v10i12.20409.

[5] -Jadczak D, Błaszczuk A, Rekowska E (2006). Effect of covering on the content of macroelements in field of basil (*Ocimum basilicum* L.) cultivated for bunch harvest. J Elementol.

[6] -Shahrajabian MH, Sun W, Cheng Q (2020). Chemical Components and Pharmacological benefits of Basil (*Ocimum basilicum*): a review. Int J Food Prop.

[7] -Qasem A, Assaggaf H, Mrabti HN, Minshawi F, Rajab BS (2023). Determination of Chemical composition and investigation of Biological activities of *Ocimum basilicum* L. Molecules.

[8] -Reichling J, Schnitzler P, Suschke U, Saller R (2009). Essential Oils of Aromatic Plants with Antibacterial, Antifungal, Antiviral, and cytotoxic properties – an overview. Complement Med Res.

[9] -Tangpao TN, Teerakitchotikan CP, Leksawasdi N, Jantanasakulwong K, Rachtanapun P (2022). Volatile Organic compounds from Basil essential oils: Plant Taxonomy, Biological activities, and their applications in Tropical Fruit productions. Horticulturae.

[10] -Aminian AR, Mohebbati R, Boskabady MH (2022). The Effect of *Ocimum basilicum* L. and its main ingredients on respiratory disorders: an experimental, preclinical, and clinical review. Front Pharmacol.

[11] -Poonkodi K (2016). Chemical composition of essential oil of *Ocimum basilicum* L. (basil) and its biological activities-an overview. J Crit Rev.

[12] -El-Beshbishy H, Bahashwan S (2012). Hypoglycemic effect of basil (*Ocimum basilicum*) aqueous extract is mediated through inhibition of α-glucosidase and α-amylase activities: an in vitro study. Toxicol Ind Health.

[13] Purushothaman B, Srinivasan PR, Suganthi P, Ranganathan B, Gimbun J, et al. A comprehensive review on *Ocimum basilicum*. J Nat Remedies. 2018;1871–85. 10.18311/jnr/2018/21324.

[14] -Darrah HH (1974). Investigations of the cultivars of basil (Ocimum). Econ Bot.

[15] Hiltunen R, Holm Y, Basil. The Genus Ocimum; Hartwood Academic Publishers: Amsterdam. The Netherlands. 1999. 10.1016/S0031-9422(01)00229-1.

[16] El-Saadony MT, ALmoshadak AS, Shafi ME, Albaqami NM, Saad AM, et al. Vital roles of sustainable nano-fertilizers in improving plant quality and quantity. Saudi J Biol Sci. 2021;7349–59. 10.1016/j.sjbs.08.032. 10.1016/j.sjbs.2021.08.032 PMC8626263 34867037

[17] Reda, El-Saadony, El-Rayes, Attia, El-Sayed (2021). Use of biological nano zinc as a feed additive in quail nutrition: biosynthesis, antimicrobial activity and its effect on growth, feed utilisation, blood metabolites and intestinal microbiota. J Anim Sci.

[18] -El-Saadony MT, Saad AM, Najjar AA, Alzahrani SO, Alkhatib FM (2021). The use of biological selenium nanoparticles in controlling Triticum aestivum L. crown root and rot diseases induced by Fusarium species and improve yield under drought and heat stress. Saudi J Biol Sci.

[19] -Dimkpa CO, Bindraban PS (2016). Fortification of micronutrients for efficient agronomic production: a review Agron. Sustain Dev.

[20] -Obreza TA, Zekri M, Hanlon EA, Morgan K, Schumann A (2010). Soil and leaf tissue testing for commercial citrus production. Univ Fla Ext Service SL.

[21] -Fernandez V, Sotiropoulos T, Brown PH (2013). Foliar fertilization. Scientific principles and field practices.

[22] -Jadczak P, Kulpa D, Drozd R, Przewodowski W (2020). Effect of AuNPs and AgNPs on the antioxidant system and antioxidant activity of lavender (*Lavandula angustifolia* Mill.) From in vitro cultures. Molecules.

[23] -Lasso-Robledo PL, Torres B, Peralta-Videa JR (2022). Do all Cu nanoparticles have similar applications in nano-enabled agriculture?. Plant Nano Biology.

[24] Win TT, Barone GD, Secundo F, Pengcheng Fu. Algal Biofertilizers and Plant Growth stimulants for sustainable agriculture. Ind Biotechnol. 2018;14(4). 10.1089/ind.2018.0010.

[25] Jowkar A, Bashiri K. GolmakaniMT.The effect of soil fertilization and foliar spray of semperflorens begonia (*Begoniasem Perflorens*) by Spirulina cyanobacterium biomass 2016.10.29252/ejgcst.8.3.65.

[26] -Tuhy L, Samoraj M, Witkowska Z, Chojnacka K (2015). Biofortification of maize with micronutrients by Spirulina. Open Chem.

[27] -Aly MS, Esawy MA (2008). Evaluation of Spirulina Platensis as Biostimulator for organic farming systems. J Genetic Eng Biotechnol.

[28] -Shawky AA, Khalifa GS, Hegazi A, ElSherif M (2023). Growth, productivity, and essential oil content of fennel plants treated with *Spirulina platensis* extract and compost tea under low nitrogen doses. GesundePflanzen.

[29] -Amer A, Shoala T (2020). Physiological and phenotypic characters of sweet marjoram in response to hydrogen peroxide and chitosan nanoparticles treatments. Sci Hortic.

[30] -Amer A, Ghoneim M, Shoala T, Mohamed HI (2021). Comparative studies of eco-friendly compounds like humic acid, salicylic, and glycyrrhizic acids and their nanocomposites on French basil (*Ocimum basilicum* L. Cv. Grand verde). Environ Sci Pollut Res.

[31] -Hammam KA, Shoala T (2020). Influence of spraying nano-curcumin and nano-rosemarinic acid on growth, fresh herb yield, chemicals compositionand postharvest criteria of French basil (*Ocimum basilicum* l. Var. Grand vert) plants. J Agri Rural Res.

[32] -Abbasifar A, Shahrabadi F, ValizadehKaji B (2020). Effects of green synthesized zinc and copper nanofertilizers on themorphological and biochemical attributes of basil plant. J Plant Nutr.

[33] Genady EA, Ahmed SS, Fahmy AH, Ashour RM. Copper sulfate nanoparticles enhance growth parameters, flavonoid content and antimicrobial activity of *Ocimum basilicum* Linnaeus. J Am Sci. 2017;13(4). 10.7537/marsjas130417.14.

[34] Jamal Uddin AFM, Rakibuzzaman M, Sabim MR, Singh K, Mahbuba S. Foliar application of copper nanoparticles on growth and yield of Chili. Int J Bus Social Sci Res. 2022; (10)1; 18–23.

[35] Lafmejani ZN, Jafari AA, Moradi P, Moghadam AL. Impact of foliar application of iron-chelate and iron nano particles on some morpho-physiological traits and essential oil composition of Peppermint (*Menthapiperita* L). J Essent Oil Bearing Plants. 2018;21(5). 10.2478/hepo-2018-0006.

[36] -Nguyen VD, Nguyen HM, Le NT, Nguyen KH, Nguyen HT (2021). Copper nanoparticle application enhances plant growth and grain yield in maize under drought stress conditions. J Plant Growth Regul.

[37] -Hernández-Hernández H, Juárez-Maldonado A, Benavides-Mendoza A, Ortega-Ortiz H, Cadenas-Pliego G (2018). Chitosan-PVA and copper nanoparticles improve growth and overexpress the SOD and JA genes in tomato plants under salt stress. Agronomy.

[38] -Pelegrino MT, Kohatsu MY, Seabra AB, Monteiro LR, Gomes DG (2020). Effects of copper oxide nanoparticles on growth of lettuce (*Lactucasativa* L.) seedlings and possible implications of nitric oxide in their antioxidative defense. Environ Monit Assess.

[39] -Wang W, Liu J, Ren Y, Zhang L, Xue Y (2020). Phytotoxicity Assessment of Copper Oxide Nanoparticles on the germination, early seedling growth, and physiological responses in *Oryzasativa sativa* L. Bull Environ Contam Toxicol.

[40] Renuka N, Guldhe A, Prasanna R, Singh P, Bux F. Microalgae as multi-functional options in modern agriculture: current trends, prospects and challenges. Biotechnol Adv. 2018;361255–73. 10.1016/j.biotechadv. 10.1016/j.biotechadv.2018.04.004 29673972

[41] De-bi -Qing-mingL, Yamamoto Z, Ichino Y, Okido R (2012). Preparation of Cu nanoparticles with NaBH4 by aqueous reduction method. Trans Nonferrous Met Soc China.

[42] -British pharmacopoeia (1963). Determination of volatile oil in drugs. The pharmaceutical press, 17, Bloomsbury square.

[43] Saric MR, Kastrori R, Couria TC, Gerie I. Chlorophyll determination. Univ. Unoven Sadu Parktikum is FiziologizeBilijaka, Beogard, Hauncan, Anjiga. 1976; 215 p.

[44] -Adams RP (2001). Identification of Essential Oil Components by Gas Chromatography/Mass Spectroscopy.

[45] Satoh K (1978). ClinicaChimica Acta.

[46] -Ohkawa H, Ohishi W, Yagi K (1979). Anal Biochem.

[47] -Aebi H (1984). Methods Enzymol.

[48] -Paglia DE, Valentine (1967). J Lab Clin Med.

[49] -Nishikimi M, Roa NA, Yogi K (1972). Biochem Bioph Res Common.

[50] -SAS/STAT (1996). Users Guide. Version 6.

[51] -Batool SU, Javed B, Sohail Zehra SS, Mashwani Z (2021). Exogenous applications of bio-fabricated silver nanoparticles to improve biochemical, antioxidant, fatty acid and secondary metabolite contents of sunflower. Nanomaterials.

[52] -Al-Huqail AA, Hatata MM, AL-Huqail AA, Ibrahim MM (2018). Preparation, characterization of silver phyto nanoparticles and their impact on growth potential of *Lupinustermis* L. seedlings. Saudi J Biol Sci.

[53] Tabatabaee S, Iranbakhsh A, Shamili M, Ardebili ZO. Copper nanoparticles mediated physiological changes and transcriptional variations in microRNA159 (miR159) and mevalonate kinase (MVK) in pepper; potential benefits and phytotoxicity assessment. J Environ Chem Eng. 2021;2213–3437. 10.1016/j.jece.2021.106151.

[54] -Shende S, Rathod D, Gade A, Rai M (2017). Biogenic copper nanoparticles promote the growth of pigeon pea (*Cajanuscajan* L). IET Nanobiotechnol.

[55] -Dimkpa CO, Mclean JE, Latta DE (2012). CuO and ZnO nanoparticles: phytotoxicity, metal speciation, and induction of oxidative stress in sand-grown wheat. J Nanopart Res.

[56] Wang Y, Lin Y, Xu Y, Yin Y, Guo H, Du W. Environ Pollutants Bioavailab. 2019; (31):80–4.

[57] -Apodaca SA, Medina-Velo IA, Lazarski AC (2018). Different forms of copper and kinetin impacted element accumulation and macromolecule contents in kidney bean (*Phaseolusvulgaris*) seeds. Sci Total Environ.

[58] Abd El-Rheem KM, Zaghloul SM, Essa EM. The stimulant effect of the spirulina algae under low levels of nitrogen fertilization on wheat plants grown in sandy soils. Int J ChemTech Res. 2015.

[59] Taha MA, Moussa HR, Dessoky ES. The Influence of *Spirulinaplatensis* on Physiological Characterization and Mitigation of DNA Damage in Salt-stressed *Phaseolusvulgaris* L. Plants. Egypt. J. Bot. 2023; 63(2):607–620. 10.21608/EJBO.2023.168006.2165.

[60] -Yanni YG, Elashmouny AA, Elsadany AY (2020). Differential response of cotton growth, yield and fiber quality to foliar application of *Spirulinaplatensis* and urea fertilizer. Asian J Adv Agricultural Res.

[61] -Anitha L, Bramari GS, Kalpana P (2016). Effect of supplementation of *Spirulinaplatensis* to enhance the zinc status in plants of Amaranthus gangeticus, Phaseolus aureus and tomato. Adv Bioscience Biotechnol.

[62] -Gharib FA, Ahmed EZ (2023). Spirulina platensis improves growth, oil content, and antioxidant activitiy of rosemary plant under cadmium and lead stress. Sci Rep.

[63] -Arahou F, et al. Spirulina-based biostimulants for sustainable agriculture: yield improvement and market trends. Bioenerg Res. 2022;10537–8. 10.1007/s12155-022-10537-8.

[64] -Pradhan S, Patra P, Mitra S, Dey KK, Basu S (2015). Copper nanoparticle (CuNP) nanochain arrays with a reduced toxicity response: a biophysical and biochemical outlook on Vigna radiate. J Agric Food Chem.

[65] -Syuhada N, Jahan MS, Nashriyah M, Khairi M, Nozulaidi M, Razali MHB (2014). Application of copper increased corn yield through enhancing physiological functions. Aust J Basic Appl Sci.

[66] -Al-Atrushy SMM, Al-Bamarny SFA (2013). Response of peach (*Prunuspersica*) cv. To foliar application of potassium and copper. J Agricultural Sci Technol.

[67] -Servin A, Elmer W, Mukherjee A, Torre-Roche RDl, Hamdi H (2015). A review of the use of engineered nanomaterials to suppress plant disease and enhance crop yield. J Nanopart Res.

[68] -Hafeez A, Razzaq A, Mahmood T (2015). Potential of copper nanoparticles to increase growth and yield of wheat. J Nanosci Adv Tech.

[69] -.Abbasifar A, ValizadehKaji B, Iravani MA (2019). Effect of green synthesized molybdenum nanoparticles on nitrate accumulation and nitrate reductase activity in spinach. J Plant Nutr.

[70] -Rizwan M, Ali S, Qayyum MF, Ok YS, Adrees M (2017). Effect of metal and metal oxide nanoparticles on growth and physiology of globally important food crops: a critical review. J Hazard Mater.

[71] -Bashir A, Rizwan M, Ali S, Zia-urRehman MZ, Ishaque W (2018). Effect of foliar applied iron complexed with lysine on growth and cadmium (cd) uptake in rice under cd stress. Environ Sci Pollut Res Int.

[72] -Hussain A, Ali S, Rizwan M, Zia-ur Rehman MZ, Hameed AA, Hafeez F (2018). Role of zinc– lysine on growth and chromium uptake in rice plants under cr stress. J Plant Growth Regul.

[73] -Masoud GAAB (2017). Effect of Plant Compost enriched with SpirulinaPlatensis Algae as a partial replacement of Mineral N fertilizers on early Sweet. J Plant Prod.

[74] -Singh S (2014). A review on possible elicitor molecules of cyanobacteria: their role in improving plant growth and providing tolerance against biotic or abiotic stress. J Appl Microbiol.

[75] -Battacharyya D, Babgohari MZ, Rathor P, Prithiviraj B (2015). Seaweed extracts as biostimulants in horticulture. Sci Hortic (Amsterdam).

[76] -Mógor ÁF, Ördög V, Lima GPP, Molnár Z, Mógor G (2018). Biostimulant properties of cyanobacterial hydrolysate related to polyamines. J Appl Phycol.

[77] -Selem E (2019). Physiological effects of *Spirulina platensis* in Salt stressed *Viciafaba* L. Plants.

[78] -El-Naggar AH, Hassan MRA, Saeid AM (2020). Growth and essential oil analysis of *ocimum basilicum*, l. plants as affected by seaweed extract and active dry yeast. Sci J Flowers Ornam Plants.

[79] -Onofrei PV, BenchennoufA, Jancheva M, Loupassaki S, Ouaret W (2018). Ecological foliar fertilization effects on essential oil composition of sweet basil (*Ocimum basilicum* L.) cultivated in a field system. Sci Hort.

[80] -Koutsos TV, Chatzopoulo PS, Katsiotis ST (2009). Effects of individual selection on agronomical and morphological traits and essential oil of a Greek basil population. Euphytica.

[81] Hassan MRA, El-Naggar AHM, Shaban EH, Mohamed MEA. Effect of NPK and Bio-Fertilizers Rates on the Vegetative Growth and Oil Yield of *Ocimum basillicum* L. Plants. Alexandria Science Exchange Journal. 2015; 36(1). doi:21608/ASEJAIQJSAE.2015.2740.

[82] Chenni M, El Abed D, Rakotomanomana N, Fernandez X, Chemat F. Comparative study of essential oils extracted from Egyptian Basil leaves (*Ocimum basilicum* L.) using hydro-distillation and solvent-free microwave extraction. Molecules. 2016;21(113). 10.3390/molecules21010113. 10.3390/molecules21010113 PMC6273689 26797599

[83] Du WC, Tan WJ, Peralta-Videa JR, et al. Interaction of metal oxide nanoparticles with higher terrestrial plants: physiological and biochemical aspects. Plant Physiol Bioch. 2017;110210–25. 10.1016/j.plaphy.2016.04.024. 10.1016/j.plaphy.2016.04.024 27137632

[84] -Nekrasova GF, Ushakova OS, Ermakov AE (2011). Effects of copper (II) ions and copper oxide nanoparticles on *Elodeadensa* plant. Russ J Ecol.

[85] -Nile SH, Thiruvengadam M, Wang Y, Samynathan R, Shariati MA (2022). Nano–priming as emerging seed priming technology for sustainable agriculture recent developments and future perspectives. J Nanobiotechnol.

[86] -López-Vargas E, Ortega-Ortíz H, Cadenas-Pliego G, de Alba Romenus K, de la Cabrera M (2018). Foliar Application of Copper Nanoparticles Increases the Fruit Quality and the content of Bioactive compounds in Tomatoes. Appl Sci.

[87] -Georgiadou EC, Kowalska E, Patla K, Kulbat K, Smolińska B (2018). Influence of heavy metals (Ni, Cu, and zn) on nitro-oxidative stress responses, proteome regulation and allergen production in basil (*Ocimum basilicum* L.) plants. Front. Plant Sci.

[88] -Hassan SM, Ashour M, Soliman AAF, Antioxidant Activity (2017). Mineral contents, vegetative and yield of Eruca sativa using Foliar Application of Autoclaved Cellular Extract of *Spirulina Platensis* Extract, comparing to N-P-K fertilizers. J Plant Prod.

[89] -Desoky EM, Rady MM, Nader MM, Mostafa NG, Elrys AS (2022). Integrated application of bacterial carbonate precipitation and silicon nanoparticles enhances productivity, physiological attributes, and antioxidant defenses of wheat (*Triticumaestivum* L.) under semi-arid conditions. Front Plant Sci.

